# Friluftsliv: the almost nearly perfect concept

**DOI:** 10.3389/fspor.2026.1705892

**Published:** 2026-02-20

**Authors:** Simon Kennedy Beames, Jørgen Weidemann Eriksen

**Affiliations:** Institute for Teacher Education and Outdoor Studies, Norwegian School of Sport Sciences, Oslo, Norway

**Keywords:** critical dialogue, equalities, friluftsliv, Norway, sustainability, three waves

## Abstract

Over the past 40 years, English-language scholarship has often portrayed Norwegian *friluftsliv* as an ideal way for people to build a simple, healthy, fulfilling, and low-impact relationship with nature. This idealized image was questioned by the first author during a conference presentation in 2024. In response, the second author addressed five critiques raised in that presentation, which led to an extended written debate between the authors in the following months. This study explores some of the supposed theoretical foundations of *friluftsliv* practices. Its goal is to offer the literature a richer and more nuanced understanding of what *friluftsliv* both is and is not. The research uses critical dialogue as its methodology: the authors' debate, structured around the five original themes, serves as the core data for interrogation. The discussion produced four main findings. First, the English language friluftsliv literature (largely authored by Scandinavians and North Americans) has depicted a certain romantic and idealized conception of friluftsliv. Second, three historical waves of friluftsliv are presented, which illustrate the changing nature of meanings associated with it over time. Third, the paper builds on the growing acceptance that friluftsliv can be difficult to define and increasingly means many things to many people. Fourth, it is highlighted how friluftsliv—just as outdoor education, leisure and tourism in other parts of the world—needs to address challenges relating to inequalities, over-consumption, and increased carbon emissions.

## Introduction and background

The genesis of this paper lies in a presentation given at the International Outdoor Education Research Conference in Tokyo in 2024. In that presentation, Simon outlined five critiques of Norwegian friluftsliv. Jørgen took issue with these critiques and wrote responses to each of them. A debate thus began, and it was decided that this could be captured and form the basis of a scholarly contribution to the literature surrounding friluftsliv and the ways in which popular, academic, and policy discourses have influenced understandings of friluftsliv in Norway, Scandinavia, and the rest of the world.

Much has been written on what is and what is not friluftsliv, and it is certainly not our intention to completely re-visit all of these points here. It behoves us, however, to outline our shared general assumptions of this term. Friluftsliv has philosophical, ideological, and normative roots that have often been associated with the fields of deep ecology and ecosophy. More recently, it has become an administrative and political tool for achieving various societal ends, such as improving public health, and has developed into a subject in the school system, as well as an academic field. Friluftsliv is thus a deeply embedded element of Norwegian history, culture and identity [see (1)]. One key prerequisite for friluftsliv, as we know it today, is the legally regulated right to roam freely in nature.

Most friluftsliv scholars place much importance on individuals feeling a certain connection with nature. For example, Breivik (2) writes of the goal being “to experience the silence, greatness and beauty in nature” (p. 2). Similarly, Dahle (3) describes how friluftsliv entails “feeling the joy of being out in nature, alone or with others, feeling pleasure and experiencing harmony with the surroundings” (p. 248). For Gelter (4), friluftsliv can be regarded as a personal “philosophical lifestyle based on experiences of the freedom in nature and spiritual connectedness with the landscape” (p. 78). Næss (5) takes this feeling of being connected to the land to the next level, in that it “cannot be separated from metaphysics” (p. 181). This suggests that friluftsliv can mean many things to many people. If we were to adopt a functional definition of friluftsliv, it is perhaps safest for us to start with that from a white paper from the Norwegian Government, “Leisure time spent being and engaging in outdoor physical activity with the aims of experiencing nature and a contrasting environment” [(6), p. 10, our translation].

This paper's title is a play on the title of the 2013 book by Michael Booth (7), entitled *The almost nearly perfect people: The myth of the Scandinavian utopia.* Booth argues that while life in Scandinavia may be enviable to many, it is not without its contradictions, problems and downsides. It is our intention to demonstrate how, particularly in the English language literature, friluftsliv is positioned as a kind of outdoor leisure utopia—but one that is perhaps not completely so. In terms of its critical stance, this paper builds on the foundational work by Gurholt and Haukeland (8), who highlight the paradoxes of friluftsliv—most notably how it falls short of its assumed ideals of environmental sustainability and social inclusion. Borrowing from Blenkinsop et al. (9), we aim to do some “philosophical house-cleaning” regarding what friluftsliv has been understood to be over time. Taking inspiration from the academic fields of engineering and art education (10, 41), and special and inclusive education (11), we decided to use our debate as data, and then use this data as a means of arriving at themes that serve our second aim of challenging and deepening our understandings of friluftsliv in the both the Norwegian and English language literature.

The paper will first outline the five critiques of friluftsliv, as presented at the IOERC conference in 2024. Next, the critical dialogue methodology used to undertake this theoretical inquiry is presented. After that, each of the five critiques is debated in turn. A discussion section then follows, where the salient themes that arose through the debate are synthesized into principal findings. The paper concludes with implications for practice and further research.

In the spirit of full transparency, we are two white, 50+, able-bodied, cisgender men who work as researchers and teachers in the field of friluftsliv at a Norwegian university. Simon grew up in North America and formed his ideas about friluftsliv from the available English language literature and frequent trips to the Norwegian mountains, while living abroad. It was only when he had been living in Norway for five years that he felt he had sufficient understanding of the literature and cultural practices surrounding friluftsliv that he, a) came to recognize that the friluftsliv he was seeing was not compatible with the friluftsliv he had read about, and b) was comfortable enough to comment on these differences in an academic forum. Jørgen was born with skis on his feet (as they say in Norway). He was raised in a typical friluftsliv-oriented family, where fishing, foraging, mountain trips and skiing were considered normal and valuable. Through academic friluftsliv studies and 20 years of teaching and research in the field, he has developed an insider-perspective on Norwegian friluftsliv. At the same time, this has elicited a rather protective attitude towards criticism from a North American scholar who argues that friluftsliv is not “all it is cracked up to be”.

## The five original critiques of friluftsliv

What might serve as a literature review in a conventional paper will be presented in the form of the five critiques presented by Simon at the tenth IOERC conference in Japan, in March 2024. Importantly, the five critiques are not presented as some kind of truth, but rather as a foil that provides a platform for eliciting meaningful debate and subsequent learning. The first critique of Norwegian friluftsliv is that it was imagined by white, able-bodied, men of privilege. While Ibsen (12) may have used the term before anyone else, he did not propose a set of its characterizing features. Friluftsliv's ideals are highly influenced by Fridtjof Nansen in particular, through a speech he gave in 1921. According to Nansen (13), friluftsliv is what young people living in urban areas should do as a break from fast-paced, modern urban life, in order to experience “primitive enjoyment” by a simple life spent in nature. Pedersen (14) explains how women have historically been marginalized in friluftsliv discourses, as there is a distinct absence of perspectives that focus on women's experiences in friluftsliv. Her later exposé on the marginalization of women argues that friluftsliv is what educated men “do” (15).

The second critique is that certain populations are excluded from what can be considered the conventional friluftsliv “story”, as outlined by Nansen (13), perpetuated by Norwegian scholars in the 1970s (82), and highlighted by Pedersen (14) and Gurholt and Haukeland (8). The most obvious example of this exclusion is Norway's Indigenous people, the Sámi, who are not part of this “nation-building narrative” (16). Non-Sami people living “nature livelihoods” (e.g., farmers and fishers) were not part of the friluftsliv story either (17). Breivik (18) suggested that there were essentially two kinds of friluftsliv: one featuring the countryside and involving hunting, fishing, and harvesting, and one featuring the cities, which featured activities such as hiking, canoeing, and skiing. Another significant population that is excluded from the traditional conception of friluftsliv is the immigrants to Norway. Some of the tensions around young people from immigrant backgrounds finding their way into this traditional world of friluftsliv have been highlighted in recent doctoral work (19, 20).

Critique number three is that friluftsliv is highly commercialized. Gelter (4) stated that friluftsliv should be non-commercial. This position resonates with writing by critics in the popular media who claim that friluftsliv has become “a fashion show” (21) and a “catwalk” (22). If we briefly move away from friluftsliv discourses and consider outdoor leisure more generally, we know that these practices are highly influenced by what is marketed to us by big business (23) and that social media influencers have a lot of sway on what practitioners think and do (24).

Friluftsliv is very competitive; this is critique four. Friluftsliv is supposed to be simple and “free” (25) and non-competitive (26), yet it has become “sportified” (27). Indeed, Breivik (26) warns against confusing outdoor recreation and “nature sports” with friluftsliv. Næss (83) took this a step further and noted that friluftsliv should be playful. More recently, Abelsen (28) has highlighted the importance of going slowly and paying attention to one's surroundings. Competitions with the self and comparisons with virtual others through technology and social media arguably run contrary to early foundational views of friluftsliv.

The fifth and final critique of friluftsliv highlighted in this conference presentation was that its practices are highly unsustainable. Næss (5) was clear that a central element of friluftsliv was not harming nature. People in Norway are well aware of the *friluftslivsparadoks*, which highlights how people's love and use of the outdoors is actually destroying it (29). In 2019, clothing and equipment purchases in Norway accounted for 1.4 billion GBP (22). Dannevig and Hall (84) outline how the biggest direct and indirect uses of energy associated with friluftsliv practices in Norway come from clothing and equipment manufacturing and travelling to sites of recreation. Perhaps even more alarming is the sharp increase in cabin (*hytte*) building (30, 31) around Norway, as this involves clearing large swathes of land and the ecologies that inhabit it—all to build luxury homes which might only be used for a few weeks a year. This is quite different from the other end of the spectrum, which features small huts without water and electricity, and which are shared by families and have relatively small ecological footprints.

The degree to which you, the reader, agree with the above critiques, is not—with respect—important at this point. What is important is how these critiques led to the generation of a pool of data that comprises our conversations about them. A description of this methodology now follows.

## Methodology

Our study is inspired by dialogical theoretical inquiries such as Blenkinsop et al. (9), in which written scholarly debate was used as data. This present inquiry uses critical dialogue as research methodology through which to undertake a theoretical inquiry. Following Jacobs' (11) work with critical dialogue, we engaged in a deep exploration of some of the putative underpinning pillars of friluftsliv practice. Our adoption of this approach to conducting research involved creating both written and online/face-to-face social spaces for discussions, where life experiences, interpretations of literature, and firmly held assumptions and beliefs could be explored, and richer understandings of friluftsliv constructed. This generative method involves using conversation to generate data (32). Jacobs leans on Bohm (33) to explain how this approach requires the actors to have a certain awareness of their own ideas, feelings and embodied responses, while also suspending judgments and suppressing impulsive reactions (p. 10). Smaling (34) explains how this kind of critical dialogue presupposes that the debaters accept that their starting points are different and that this process will feature an element of “uprooting” and willingness to let go of one's long-held convictions. Critical dialogue thus emphasizes “clarifying, understanding and respecting, and also making communicable, differences in opinion” [(34), p. 12]. The extant literature focuses both on conversations between some kind of facilitator and participants [see (11)], and on interpreting discussions between the two authors [see (10)].

Ultimately, this critical dialogue approach rejects post-positivistic paradigms (35) and thus finds itself in a similar paradigmatic space to collaborative autoethnography (36) and narrative inquiry (37). Through bringing the element of debate into our philosophical interrogation of the literature, our reflections, interpretations and judgments became part of the material under scrutiny (38). This enabled us to bring some methodological plurality to the friluftsliv corpus of literature by eschewing “the constraint of prescriptive methodological parameters” [(35), p. 450]. Through participating in a form of intertextual dialogue [see (85)], our ultimate, and perhaps utopian, goal was to arrive at what Anderson (39) calls “theoretical illumination” (p. 388).

The actual method involved four phases. Phase one featured three steps, lasted for four months, and involved sending written comments, challenges and rebuttals to each other. First, Jørgen responded to each of Simon's five critiques. Second, Simon addressed the responses. Third, Jørgen then responded to these. Each of these responses involved bringing in new literature to add detail, nuance, and depth to the debate. Phase two involved debating our positions face-to-face over a two-day period. This phase focused on going more deeply into arguments that were not adequately resolved in phase one and then refining the text more generally. We left that block of time with an early draft of the background to our debate, an outline of a methodology section, 10 pages of written debate notes that needed to be reduced into digestible and shareable text, and a list of government reports, speeches, book chapters, magazine articles, and journal papers to be examined and integrated, as needed. Towards the end of our time at this writing retreat, we scrutinized which arguments and sources could be added to our debate, and which could be removed, and emerged with a rough version of a paper that remained littered with comments boxes of tasks to do.

These first two phases resemble a spiral that can be likened to the hermeneutic approach used by Patterson et al. (40), where the researchers continue examining the text, and their interpretations of it, until they are convinced that it serves the aims of the investigation. Much of what emerged in these first two steps could be described as what Guyotte and Sochacka (10) call “productive tensions”, as they “bring forth our respective and often differing theoretical perspectives” (p. 2). These authors advise against trying to avoid conflict and embarrassment, and explain how this approach “allowed ourselves to enter vulnerable spaces, so that we could contribute to the conversation as our most ‘flawed’ selves” (p. 5). Trust in one's co-author(s) is of the highest importance. As two colleagues who already work closely together, this trust permitted us to challenge each other's deeply held assumptions and biases, which are very naturally shaped by our contrasting cultural upbringings, without risk of humiliation.

Phase three of the process was simply to write a coherent story that outlined the rationale, methodology, key findings, and implications for practice, policy and further research. At no point did we use any kind of thematic analysis or phenomenological reduction of text to themes. Rather, we used our unpolished version of the paper from phase 2—which had five predetermined themes—as the basis from which to write this refined draft. Phase four involved sending our draft to trusted colleagues—some of whom played a dominant role in our text, and others with an appropriate amount of emotional distance and knowledge of the field, who would be able to critique the paper in a more objective manner.

The constant back-and-forth nature of our debate brought with it a form of in-built “procedural validation” [see (41)]. This kind of data verification, combined with the peer-review afforded us by our colleagues [see (42)], was employed to lend the findings a higher degree of trustworthiness (43).

In the next section, the findings are presented as edited representations of the essence of our written debates and verbal exchanges. This reduction of text and amalgamation of text involves making certain methodological choices about what is central to the aims of the paper, given the word count restrictions of a conventional journal article. We have endeavoured to make our processes within this academic inquiry as rigorous as possible. Stake (44) once noted that, “It is my integrity as a researcher that I beg to be recognized, that my interpretations be considered” (p. 76). In the very same way, we ask that readers give our findings similar credence.

## Findings and discussion

In this section, we present the findings and discussion combined around each of the five critiques in section 2, which originally came from Simon's conference presentation.

### Friluftsliv was imagined by white, able-bodied, Men of privilege

Jørgen. Are you criticizing the fact that the founders were rich old men, or are you critical of the consequences of their actions? And who are you thinking of when you refer to “white, able-bodied, men of privilege”? If you have Fritdjof Nansen in mind, it is important not to forget that friluftsliv flourished before Nansen gave his famous friluftsliv speech in 1921. The DNT (Norwegian trekking association) was formed in 1868 (yes, by five white, able-bodied, men of privilege), but it was caused by the need to organize and facilitate for the growing herds of people that wanted to experience the forests and mountains in their leisure time. In the early DNT yearbooks (as early as the 1870s), one can find accounts of women in the mountains, along with many calls for women to participate (81): “everyone to the mountains” was a rallying cry and the DNT was open to female members from day one. Indeed, in the DNT cabin books from the 1880s, one can see that 20%−30% of the visitors were women (81).

Slagstad also explains how in 1886, inspired by Dr. Edvard Bulls' speech about women's health and well-being, the Norwegian Women's Rights Association positioned friluftsliv as an arena for equal participation and influence in society. In the same year, a new style of outdoor clothing called “the reform outdoor dress for women” was introduced, which enabled women to use their bodies more freely during outdoor activities; corsets and lace were replaced with fabrics that were deemed more hygienic and comfortable, and this new dress, designed in Sweden, was sold in the leading fashion store in Oslo (Kristiania) (81). Slagstad notes that even in those early days, friluftsliv was “a field that provided space for physical bodily expression for women too” (p. 95). My point here is that friluftsliv evolved in a time when masculinity influenced all parts of society. It is therefore relevant to question if friluftsliv was worse than other comparable areas like culture, arts and politics. Indeed, when compared to competitive sports, friluftsliv arguably gave women more possibilities and more opportunities for participation.

Simon. Nice argument! But I still maintain that the white, able-bodied men of privilege established some friluftsliv structures and practices that served their own needs and interests. In general, I think of Nansen's ideas in the early 1920s that were perpetuated by Faarlund and others in the 1970s, 80s, and even 90s. For clarity, when I say “privilege”, I mean those who have received certain advantages, such a high level of education, money, favourable access to power or resources, and—with importance to our discussion—leisure time.

As Gurholt (15) argues, friluftsliv can historically be seen as a men's playground. Back in 1999, she highlighted how, within the field of friluftsliv research (as opposed to practice), women's perspectives have been largely absent. She posits that research, management, and didactics within this field are based on an implicit, yet unspoken, male perspective, and I agree that there exists a certain cultural dogma based on masculinity within the field of friluftsliv. Singsaas (45) notes with reference to Gurholt, that many dominant understandings of friluftsliv primarily represent men's experiences, as reflected in the literature and thus contribute to their reproduction. I think my question, What might friluftsliv practices look like if they had been shaped by women?, remains reasonable to ask.

Jørgen. Here, I think we need to bring a little more precision to our debate. Following Pedersen (14), I agree that women's perspectives have been largely absent in early research. But it is important to note that she was referring to the initial period of research, between 1970 and 1995. This period was heavily dominated by male academics, many of whom paid little to no attention to female perspectives on friluftsliv. After Gurholt's PhD in 1999 (the first on friluftsliv), the stage changed. Over the last 25 years, a further 12 PhDs on friluftsliv have been completed by Norwegian women, and in my opinion, the masculine imprint on friluftsliv research and the discourses within it are steadily fading.

I have a bigger problem with your second question, however: *What would friluftsliv practices look like if they had been shaped by women?* There is a fair amount of evidence to suggest that many outdoor practices were indeed influenced by women. Pedersen (14) writes about how women in the north of Norway have been engaged with, and developed, several nature-based skills and practices as part of their daily activity. Vingdal (46) describes similar perspectives in her book about the women working on summer farms in the Norwegian mountains. What is typical in these descriptions is that the women were not very concerned with the label “friluftsliv”. While they engaged in nature-based activities that were necessary for the family's well-being and income, they contributed to developing practices belonging to the friluftsliv sphere. So, women have not been insignificant in the development of Norwegian friluftsliv practices, both in the earlier days and in more contemporary times.

If we turn to the recent reports on friluftsliv participation patterns in Norway between 1974 and 2021 (47), we can see that participation levels are more or less equal between the genders. Men are overrepresented in hunting and fishing, while more women take part in walking, picking berries, and picking mushrooms, for example. When it comes to hiking and skiing, the numbers are pretty much the same. Amongst youth, the number of girls involved in friluftsliv is increasing: a slightly higher number than boys between ages 6 and 15 (except for fishing, cycling, and long hiking trips) (47). So, even if the field was developed by men, and has been influenced by male perspectives and figures, it looks like friluftsliv practices have developed into something that women really appreciate and even thrive within.

Getting back to our discussion on influential men and Norwegian friluftsliv, note that while Ibsen is often associated with the origins of the word “friluftsliv”, his role in the development of friluftsliv as a field of practices and discourses was probably not very important. He used the word in one poem (1859), yet there are no references to this poem in academic writing and government policy before the 1970s. The initial period (second half of the nineteenth century) was followed by a new dimension of friluftsliv that emerged around the turn of the 20th century. The polar explorers, with Fridtjof Nansen as a central figure, laid the groundwork for friluftsliv to become part of a national identity project. This momentum reached its peak in 1911 when Roald Amundsen, relying on robust friluftsliv skills, reached the South Pole, triumphing over “the British Empire.” This era, when many states were focused on nation-building through exploration and exploitation, finds its roots in Christianity with Genesis, which Heibert (48) and Holland (49) explain has shaped Western values of human (but largely coded as male) mastery, and control over nature.

In this same light, friluftsliv was increasingly seen as a prerequisite for masculine endeavours in nature. During the first half of the 20th century, fur hunters like Helge Ingstad and the resistance movement during the Second World War, often operating in the forests and mountains, added more masculine values to the field of friluftsliv. I agree that women were not very visible in these contexts. These privileged white men probably created a foundation for Norwegian friluftsliv and national identity, and have, in different ways, influenced the development of the influential discourses that came to prominence in the 1970s.

Regarding Nansen, it's pretty clear that he was speaking to both men and women; in his speech in 1921, he didn't differentiate between the two. Still, I am unsure how much these early ideas have actually influenced the practices of ordinary hikers, skiers, and mushroom pickers today. I think a large proportion of modern-day friluftsliv practices are undertaken by people with no clue about the origins of the word or the academic and political development of the field.

If we move away from these early characterisations of what is friluftsliv (such as in Nansen's (13) speech), to the development of friluftsliv discourses, it was only in the 1970s that this really gained momentum. Faarlund, Næss, Kvaløy Setereng, and Breivik were part of this, and had great influence on the academic development of friluftsliv, as well as on the development of friluftsliv within the political sphere. This latter point is supported by Horgen (50), who explains that the “idea of friluftsliv as nature-friendly travel, characterized by simple equipment and the absence of competition, ultimately became hegemonic. This prevailing notion gained significant influence, even shaping the authorities' understanding of friluftsliv” (p. 38).

Note that these were many of the same actors that Gurholt was implicitly admonishing for their exclusion of women's perspective on friluftsliv. But when it comes to answering the question, “What is friluftsliv?”, in public consciousness today, I think the most notable contributions have come from academic research post 1995, conventional and social media, big national friluftsliv organizations, and public administration bodies (e.g., directorates and ministries that have published parliamentary reports, subject plans for schools, conservation plans), and so on.

Simon. This makes sense, as much of what you cite (e.g., friluftsliv organizations, parliamentary reports, school curriculum) is published in Norwegian and therefore not as accessible to those who do not read Norwegian (especially in pre-GoogleTranslate times). For us foreigners, what friluftsliv “is” (or was) was articulated by a relatively narrow group of Norwegian men who found a voice in some English medium outlets, such as *Wisdom in the open air* (51) and *Nature first* (52). I know that with the latter, Henderson was trying to articulate a kind of education that he had encountered in Norway that was about more than mere outdoor activities. He wanted to lift-up a very deliberate way of living at home and travelling through the world that involved fostering deep and caring relationships with self, others and place.

Jørgen. I think that foreigners like you, through Henderson and Vikander's book (among others), have gotten the impression that the ideological basis for outdoor life (with a background in deep ecology and ecophilosophy) is a strong and present feature in today's friluftsliv in Norway. However, I think this is over-blown. The philosophical underpinnings definitely did reach the surface, but to claim that friluftsliv is the Norwegian way of embodying the philosophical ideals of deep ecology and ecosophy is an opinion that is strongly held only within a small circle of elite friluftsliv academics, not least those who contributed to *Nature First*.

We also mustn't forget the role that the media has played in the development of popular friluftsliv practices. In the last 30 years, magazines such as *Vi menn*, *Fri flyt*, and *Ute*, as well as countless television programs, such as *71 degrees north* and *Monsen på villspor*, have collectively had a massive influence on the Norwegian public. There has also been a shift towards portraying the sexes more equally, and children have also been given a more central place. See for example, *Villmarksbarna*, which follows the outdoor adventures of three children (two sisters and one brother) in the north of Norway.

### Certain populations are excluded from the conventional friluftsliv “story”

Jørgen. I believe *not being included* is a better way of stating this than *being excluded*. I don't think anyone was intentionally being excluded. But I think you are right about this—especially regarding the Sami people, as a deeply nature-oriented culture, developed through generations, have effectively been ignored from the friluftsliv story. But it is also possible to rewrite the question: Why should the rich nature-based culture and heritage of the Sami people be reduced to something inside the label of friluftsliv, a label invented by city-people fifty to 100 years ago? Skille, Pederson and Skille (16) argue that this smells like something that has been described as post-colonialism. I don't think the Sami people had, nor have, any wish to be included as anything inside the friluftsliv sphere. I think they want to be recognized for what they are: an Indigenous population with deeply rooted nature-based practices.

However, with regard to the part of the Norwegian population who have historically subsisted on and from nature, whether it be farmers, hunters, milkmaids in the mountain pastures, loggers or fishers, the picture is a bit different. People working on the land and sea were outside the “normal” scope of friluftsliv activities that was inhabited by the hikers and skiers, whose picturesque descriptions of their outings captured the public imagination. But this story is not without exceptions. In 1939 and 1943, two notable books were published: *Friluftsliv—from Mountain and forest, from sea and land* (volumes 1 & 2) [Blix (53), Blix (54)]. In addition to the expected descriptions from city people's mountain trips, the chapters highlight many stories from foresters, cartographers, sailors, and fishermen, who had “dutiful”, daily encounters with nature through their work. It is thus possible to argue that such encounters with nature, back in 1940, were actually included in the “friluftsliv story”, while being part of a more widely accepted understanding of friluftsliv.

Your point about friluftsliv and immigrants is a different one. Before 1970, more people wanted to emigrate than immigrate, and it was not before the late 1980s that we saw a marked increase in the number of immigrants coming to Norway (55). With increased immigration, the need for integrating newcomers into Norwegian society was placed on the political agenda, and friluftsliv was considered to be a suitable arena for this task by the head of the national friluftsliv organization, Norsk Friluftsliv (56), and by the government (Meld. St. 18, 2015–2016). I would argue that including immigrants in friluftsliv has been an important part of the “story” since the first academic attempt in the middle of the 1990s [see (57)].

Simon. Yes, and as with the first critique, it is vital that we as academics recognize more universally that these specific groups were not originally part of the friluftsliv story. Can't we all simply accept this in a more obvious and humble manner?

Jørgen: We should be humble, but at the same time, I think it is our duty as academics to critically question the narratives that are created to fit someone's aims and goals. The Sami people are unquestionably defined outside the friluftsliv story, and maybe for reasons that are well and good. Those who subsisted on/from nature are probably more recognized as part of the story than the early friluftsliv academics emphasized. When it comes to the immigrant population, well, they entered the story after it had originally been established.

Simon. I understand that early conceptions of friluftsliv did not set out to marginalize or exclude those who moved to Norway from other countries and cultures. My point remains the same, however, in that many immigrants in Norway simply do not see themselves within the idealized friluftsliv culture, that was first conceived over a century ago, and which involves doing specific activities, with particular clothing and equipment. Recent statistics state that 35% of Oslo's inhabitants are immigrants or have parents who have immigrated (55). In terms of friluftsliv participation, people with lower socioeconomic status are underrepresented, and a disproportionately high number of immigrants belong to this group; young people with university-educated parents participate more than those with less educated parents; people with physical and mental disabilities are underrepresented; and immigrants from Africa and Asia are underrepresented, with their children being the most notable examples (47). My main point here is that friluftsliv practices may not be as universal as some people assume them to be.

Jørgen. I think you're right. The will to include immigrants into friluftsliv is definitely present, but to what degree we as a society are successful in doing this is another question. In Norway today, friluftsliv is considered a central arena in which to integrate immigrants—especially those who are younger. And the logic is simple: in order to be integrated into society, one must understand and be exposed to national culture, and friluftsliv is considered to be an important vehicle for this. Anderson (20) states this very clearly in her claim that “friluftsliv has become a symbol of ‘Norwegianness’” (p. 7). This logic is visible in government policy (6), and friluftsliv is even a part of the formal “introduction program” for refugees coming to Norway (58). If you look further in Gurholt, Torp & Eriksen (59), and their work on friluftsliv and youth in Oslo, you will see that a huge amount of the financial resources directed towards friluftsliv go to this group. Indeed, in 2018, more than 50 million Norwegian kroner was earmarked by the Norwegian Government to increase immigrant participation in friluftsliv (60). That said, recent research has problematized the credibility of this logic. Broch's (19) findings highlight the fact that some young people with darker skin colours and who have an immigrant background feel stigmatized and uncomfortable when practising friluftsliv. Walseth and Kahn (86) conducted a study in tertiary education in Norway, and their findings indicate that friluftsliv is characterized by whiteness, and explain how this contributes to the marginalization of students from minority backgrounds. In her PhD dissertation, Anderson (20) concluded that using friluftsliv as an arena for integrating refugees into Norwegian society is much more complicated than the responsible authorities seem to believe.

Simon. From governmental policy and financial perspectives, it is commendable that Norway spends so much money on supporting immigrants through friluftsliv. It would appear, however, that these initiatives have not been especially successful. So, while there are all kinds of opportunities for people without a long Norwegian heritage to participate in outdoor activities, I would argue that most outdoor spaces beyond the barbecue pits in lakes nearby to Oslo are very white. Irrespective of how much money is being thrown at inclusive friluftsliv practices, a limited amount of literature in Norway [e.g., (19)] and a lot in the US [e.g., (61, 62)], suggests that many people with immigrant backgrounds simply don't feel comfortable and welcome in certain outdoor spaces, because they do not see people who look like themselves or they do not perceive themselves to possess high enough levels of outdoor recreation skills. Since we are white and have certain unconscious competences and friluftsliv literacies, this is hard for us to know. The “Young in Oslo” research highlighted that there were two main barriers for young people getting involved in friluftsliv activities. The first is their family's financial constraints, and the second is their lack of skill due to less socialization into outdoor activities with their family, insufficient opportunities to develop friluftsliv skills at school, and limited organized activities during leisure time in the local environment (59).

Jørgen. Having clearer insights as to these obstacles is helpful, but the relatively lower involvement of children and young people with immigrant backgrounds persists. Having said that, other things going on that I believe have good potential. Friluftsliv has, since 2020, a more prominent and “enlightened” position within the most recent version of the national school curriculum. Even if friluftsliv, as a term, has been replaced with the core element “outdoor activities and nature travel” (63), the content is deeply anchored in common friluftsliv practices. The name-change has been taken precisely to respect the Sami people's way of being in nature. Indeed, several Sami nature traditions are featured very clearly in the learning outcomes, but not as friluftsliv. For me, this is a way to include different nature-based practices and to show the Sami people the respect they deserve.

Simon. It's also good to see the recent attention to the Sami in the academic literature. Authors such as (64) Krempig and Enoksen (64) highlight the importance of involving Sámi traditional practitioners and incorporating Sámi practices across the school curriculum.

### Friluftsliv is highly commercialized

Jørgen: First, I think it is necessary to attain an agreed understanding of what we understand by your term, “highly commercialized”. You use Gelter (4) as your source on this topic. And this is probably a good place to start. He argues that “a strong commercialization creates a never-ending flow of new consumption-lifestyles for outdoor recreation. Activities and equipment now overshadow the original goal of friluftsliv to be close to nature” (p. 80). For Gelter, commercialization leads to two undesirable consequences: new outdoor activities, which hamper our possibilities to have deep experiences with the landscape, and the massive consumption of equipment that enables us to use nature as an arena for testing oneself and the equipment. If I understand your point correctly, this is what you have in mind when you present commercialization as problematic?

Simon: Yes, that's right. Perhaps a better term for me to use is the *consumption* of outdoor clothing and equipment. Interestingly, in a 2025 book chapter that just came out, Sandell and Öhman (65) recount how back in the 1980s in Sweden, a more radical, pro-environmental form of friluftsliv called *Argaladei,* raised concerns about society's obsession with “materialism and commercialism” (p. 21). Their discussion argues that consumption was and still is a big problem within society—and friluftsliv is no exception.

Jørgen: What is interesting is that, even if Gelter and Sandell and Ohman argue that commercialization is something we don't want in friluftsliv, I don't think anti-commercialization is very widespread in friluftsliv. Over the years, Faarlund has been critical of commercialization in many ways, but even here it is possible to find a pretty big paradox. His own magazine, *Mestre Fjellet* in the 1970s, was full of advertisements for outdoor equipment. And speaking of commercialization, one of Faarlund's life projects was training mountain guides for commercial guiding in the mountains. So, even if Faarlund is particularly puritanical himself, his impetus for commercializing friluftsliv was quite strong. Today, universities in Norway are educating students to work within the steadily growing field of market-based nature tourism, and there are few critical voices.

Let's get back to outdoor clothing and equipment, where my main point is that not all development is negative. Take the early explorers, for example; they were very keen to use optimal equipment, and Amundsen arguably won the race to the South Pole because of his attention to this. Nansen and Amundsen would probably be delighted to see how outdoor clothing and equipment have evolved over the years.

Simon. I don't disagree with any of this. However, can you imagine what Nansen, Næss and Faarlund would think of the absolute glut of outdoor “stuff” that people can buy online and in stores? I think they would be disgusted at how much money, material, energy and labour is thrown at a leisure activity that, according to Næss (5), was supposed to embody the mantra of “simplicity in means, richness in ends” [(66), p. 7 and (2)]. Earlier in this paper, the vast sums of money that Norwegians spend on outdoor clothing and equipment purchases was highlighted (22). The whole gear manufacturing process is, I think, a key critique of contemporary friluftsliv, as it seems highly aligned with society's consumption practices in many ways, and which would probably have Næss, Nansen and Kvaløy Setereng rolling in their graves!!.

Jørgen. Maybe not Nansen! But I see your point. The problem is maybe not commercialization *per se,* but the over-consumption that follows—especially with products that are not considered sustainable. The commercialization part of it can maybe also be seen as something positive for friluftsliv, with its focus on the benefits of an active lifestyle and as an impetus for recruiting new participants to friluftsliv, who do not have a strong tradition of it in their family.

These days, you see people out hiking who seem more interested in showing-off than in experiencing nature. At the same time, however, I notice that some people are very deliberate about not wearing trendy clothes while outdoors. If we agree that outdoor clothing itself has become cool streetwear (the “Patagoochi effect”), then you could ask whether outdoor clothing as fashion is worse than any other fashion brands. With the green wave washing over clothing producers in the outdoor industry, one could argue that the outdoor industry actually contributes to a more sustainable fashion scene—especially considering the durability of the clothing and the increased focus on natural fibres.

### Friluftsliv is very competitive

Jørgen. Unsurprisingly, I disagree with you! But, before we examine the situation today, I believe it is necessary, again, to turn to some historical sources. Horgen (67), in his 2022 paper, analyses the Norwegian Trekking Association's yearbooks. He found that between 1869 and 1920, the term friluftsliv was hardly used. After Nansen (13) distinguished in his 1921 speech between friluftsliv as deep encounters with nature, as opposed to the focus on competitiveness in sports, the authors of the yearbooks still did not use friluftsliv to describe their experiences in nature. It was more normal to use the terms idrett/sports. The rejection of competition in friluftsliv first emerged in the late 1960s alongside deep ecological norms (68). So, one could argue that if friluftsliv (or parts of it) is competitive today, it is not very contrary to early foundational views before the 1970s.

Simon. While I totally agree with your point that many people distance themselves from the fast pace of contemporary society by finding quiet and solitude in the woods, there are also many who are highly driven and use technology and social media to “compete” with themselves and others through their training and who compete in a way with “online others”, through the internet-based curation of their public outdoor identities and by comparing their Strava times for a given route [see (23, 69)]. What I find especially fascinating is the outdoor activities that do not look competitive, but which really are.

Jørgen. I guess we also should add some comments on the subjective and the nature-connectedness elements [e.g., from (2)] when trying to draw a line between friluftsliv and competition. For example, what do you want to achieve while participating in an outdoor activity? Is it to win, perform, set new records, or is it “to experience the silence, greatness and beauty in nature” [(2), p. 2], even if the activity is undertaken with the same equipment, in the same places, with the same people. My point is, with reference to Breivik, that our subjective mindset during outdoor activities will decide if our aim is competitive or not.

Faarlund (70) had an interesting comment on this, where he discussed climbing (p. 67). He argued that climbing could be understood as friluftsliv (the Scandinavian way) and as sport (the way it has developed in the Alps, Japan, the UK, and the USA). He also argued that parts of orienteering, rowing, running, skiing, paddling and horse riding should be excluded from the friluftsliv family, when competitions are part of the activities. These arguments were introduced in relation to a process where they tried to define which activities should be included under the friluftsliv umbrella, and which should not. That said, it could be argued that all kinds of activities—even if they were part of some kind of competition—can offer possibilities for participants to have intense experiences in and with nature. Following Næss (5), on those grounds, these activities could be called friluftsliv, as they elicited a certain subjectively held depth of experience.

You claim that today's outdoor life is not compatible with earlier ideals, but historically speaking, what were these early ideals? If one goes to the period before Nansen's speech, there were all kinds of outdoor competitions: there was a race to cross Greenland, a race to reach the poles, being the first to climb the biggest mountains, to catch the biggest fish, and so on.

I still go back to friluftsliv having elements of competitiveness as not being anything new; it has arguably been part of friluftsliv values for the last 150 years. To say that friluftsliv is particularly competitive today is, in my opinion, not very precise. Large parts of those practising friluftsliv deliberately distance themselves from the competitions, and take care to go slowly, stay closer to home, eschew regimented ways of being, and avoid using technology. At the same time, you can see that countless adventure-based competitions have adopted elements from outdoor life. However, these competitions are probably perceived by most people more as sports competitions than as outdoor activities [in line with (26)].

Simon*.* I can accept that, historically, there has been a fairly strong element of competition in some aspects of friluftsliv. It appears that these discussions of friluftsliv are located within an ever-broadening scope of outdoor leisure time. On one hand, there are those who are super-deliberate with how they are using the outdoors as a vehicle for slowing down and escaping their competitive worlds, as described by Lynch and Moore (71). However, some are highly competitive and use outdoor landscapes as sporting arenas, and have effectively brought their fast-paced modern society with them into the woods. Buckley's (72) work argues that outdoor adventure activity participants seek risk, challenge, and competitive engagement with the environment and themselves.

Jørgen. This notion of outdoor activities as a training arena not being part of friluftsliv is somewhat reductionist. I think many people go out into nature to improve their fitness. Even if the goal is physical training, parts of the outing can still be regarded as friluftsliv. For example, you can come to places where you stop to enjoy the beautiful colours of nature, you can get curious about an animal track, or you might hear a bird chirping that makes you stop and listen some more. My point is that there are grey and overlapping areas related to training, competitions and friluftsliv. The lack of literature on this topic suggests that this nexus of human competition with oneself, others, and the natural world, and friluftsliv as a leisure space for reflection and contemplation in, and connection to, nature is ripe for academic inquiry.

### Friluftsliv practices are highly unsustainable

Jørgen. In my opinion, this is the biggest problem with friluftsliv today. And it is complex! It is reinforced by nature-based tourism and is stimulated by social media and made possible by a wealthy economy. It could be possible to argue that long, challenging journeys and fancy equipment are a part of the friluftsliv story, but what we see today is something totally different. And it is impossible to simultaneously ignore it and legitimize it. People travel from all around the world to visit Trolltunga, Lofoten and Preikestolen. And Norwegians travel all around to hike, climb, go skiing, and so on. This may be considered as tourism, and not friluftsliv, but the lines between them are thin and vague. Crucially, the consequences of these actions threaten nature, and in the long run, friluftsliv.

Simon. Right, so here we are more of the same mind. The carbon emissions that come from friluftsliv-related travel have increased in staggering amounts over the last 100 years. Indeed, Aritza and colleagues (87) estimate that friluftsliv practitioners generate between 3.4 and 4.7 tons of CO2 equivalent emissions annually from their friluftsliv activities. This amount represents approximately one-fifth of the average Norwegian's total yearly greenhouse gas emissions are more than twice the emissions boundary required to limit global temperature rise to 2 degrees Celsius. The authors claim that Norwegians live in the “façade of green lifestyle” that obscures a rather substantial carbon footprint. The classic work done by Aall and colleagues (73) highlights how leisure consumption is growing at an even faster rate than everyday societal consumption.

Of course, the pioneers were travelling to faraway places, but here we are discussing everyday or at least “regular” friluftsliv practices. People in the global north have much more disposable income than they did 100 years ago (even since the early 1970s), and this is used to drive all over the country and to fly overseas—all in the name of friluftsliv. It wasn't like that until after the Second World War in any country, so this is not a critique aimed squarely at Norwegians.

Jørgen. I'll give you that, and this relates to my comment above, where the friluftsliv paradox of outdoor-related actions that are actually damaging the outdoors is hard to overcome. However, at the same time, daily friluftsliv practices can connect people to local areas. One could say that, in a global perspective, friluftsliv-related actions such as buying new equipment and clothing, driving and flying to remote places, have developed into a phenomenon that is hard to defend. From a more local perspective, however, friluftsliv can be important for developing attitudes and actions related to improving nature conservation.

Simon. This last point is somewhat controversial, isn't it? Sobel (2008) and others claim that we need to play in outdoor spaces in order to love them and then take care of them, but some—including our colleague Jannicke—critique this position [see (74)] by arguing that interacting with and loving nature does not necessarily lead to increasing a person's sense of active environmental stewardship. I'll add that even though Dannevig and Aall (75) have rather dated data, their analysis shows that in Norway, the highest costs to the environment come from building cabins, heating cabins, driving personal vehicles to and from outdoor sites, and manufacturing outdoor equipment.

Jørgen. Moving on to another element of your fifth critique, we need to discuss the case of these large “cabin fields” being built. As you know, private cabins are a large part of Norwegian national identity [see (76)], yet this traditional narrative of the small *hytte* in the forest or on the coast has arguably become outdated (77). Today, more than 450,000 private cabins have been constructed in wilderness areas across the country, and 70,000 of these have been built in just the last 10 years (78). These large, house-like cabins have placed a huge footprint on vulnerable landscapes and have become the centre of a large controversy in Norway—one that probably deserves its own paper.

Simon. So true! Readers who want to explore this topic further can look at work by Xue et al. (30) and Iversen et al. (31), for example, as they explore wider issues related to government policy, environmental degradation, impacts on local communities, and carbon emissions related to building these new fields of cabins. Sustainability was probably not on the radar of most friluftsliv practitioners until 1970, when Naess and Kvaløy Setreng (79) spearheaded the resistance movement against the damming of the Mardøla River. In the period that followed, they (together with Faarlund) worked to position friluftsliv as a set of practices that were rooted in living lightly on the earth.

Jørgen. Yes, exactly. Thus, it is arguable that friluftsliv's origins never featured explicitly pro-environmental aims.

## Conclusions

The five original critiques may have focused on Norway, but they reflect a similar story of outdoor practices in other places: much of what counts as proper outdoor education, leisure and tourism in regions all around the globe was developed by white, able-bodied, men of privilege; outdoor practices often exclude large parts of the population—usually marginalized people, such as people of colour, Indigenous people, the poor, immigrants; outdoor practices are highly commercialized and driven by big businesses and their social media influencers; outdoor leisure practices can be very competitive; and outdoor equipment and clothing manufacturing, along with travel to and from outdoor play sites, are damaging to the environment.

While the majority of this text has focused on what is wrong with friluftsliv, there is much that is very positive and which should be celebrated. For example, simply having the “right to roam” (*allemannsrett*) and excellent public transport makes beautiful green spaces accessible to most people in Norway. The number of girls involved in friluftsliv is steadily increasing and there are individual social media influencers who are challenging what a “normal” friluftsliv person looks like. Some big outdoor clothing companies are becoming very mindful of offering opportunities to repair equipment and are manufacturing products that are “nature-friendly” (e.g., with 100% used material). And many Norwegian organizations are very active in promoting cultural integration through outdoor activities, with the Norwegian umbrella body, Norsk Friluftsliv, leading the way.

It would seem that there is no “one” accepted set of assumptions that characterizes Norwegian friluftsliv. Our examination of the germane literature has revealed how public intellectuals, scholars, and politicians have tried to define the term “friluftsliv” for the good of the Norwegian people, as a collective and as individuals, but have failed to find consensus. Borrowing from Johnston and Percy-Smith's (80) work on conceptualizing social capital, friluftsliv is a very difficult term to capture in a precise manner, as “the closer one gets to it, the more slippery it seems to be” (p. 322). Attempts to address the slipperiness of the word have arguably led to certain influential texts in Norwegian, and later English and German, which have positioned friluftsliv practices as a romantic and idealized construction founded on principles of deep ecology.

While we have attempted to undertake a critical “deep dive” into the key assumptions underpinning friluftsliv practices, this inquiry has raised questions that can arguably be asked of all kinds of outdoor education, leisure and tourism practices, in whichever country or region they may take place. These are:

How can outdoor education and leisure practices be made to feel normal, natural and good for all kinds of people and their varying needs?
How can equipment and clothing consumption be diminished?
How can carbon emissions from travel to far-away sites be minimized?
How can big business, individual influencers, and educational organizations shape a greener, more equal friluftsliv?

**Figure 1:**
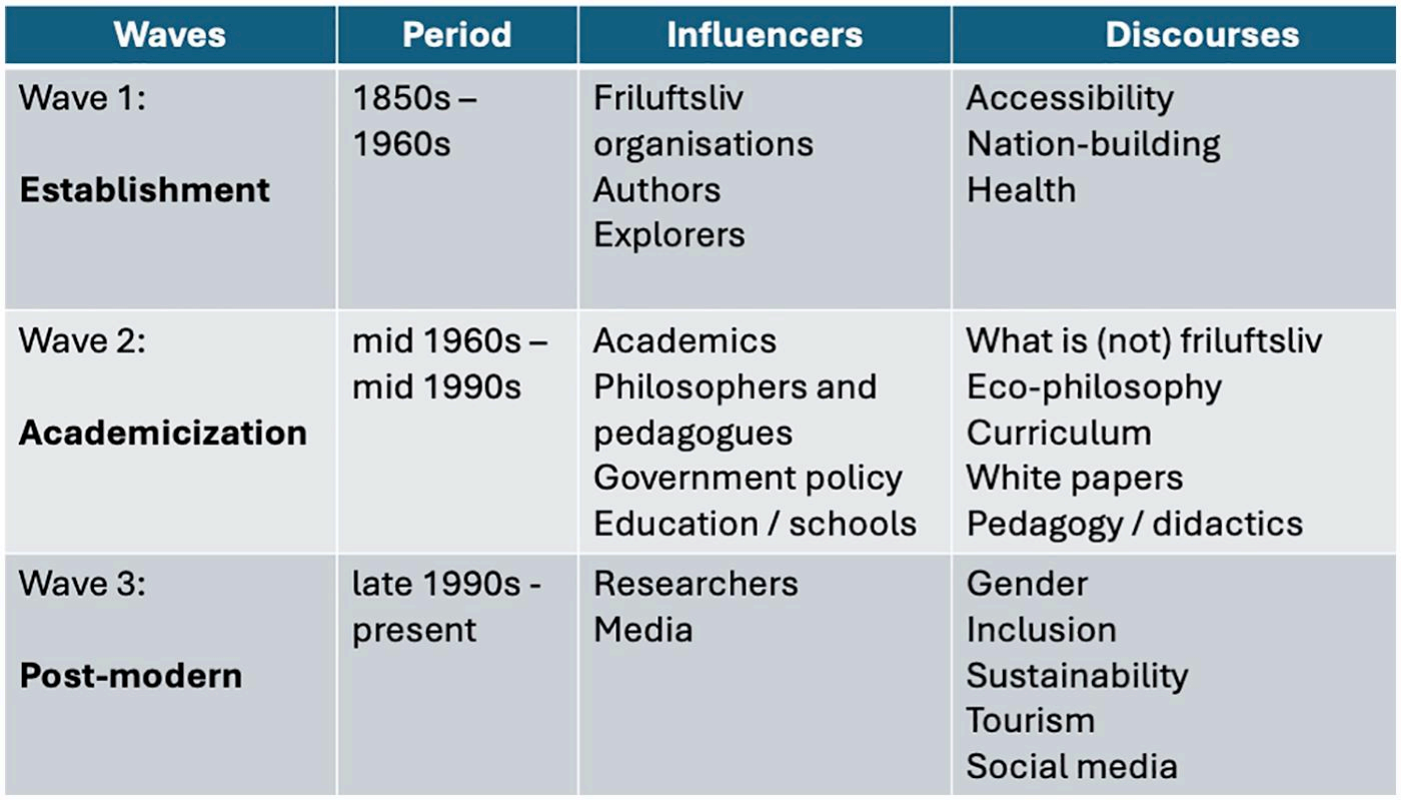
Three historical waves of friluftsliv.

As we attempt to bring this discussion to a close, we see the conceptual beginnings of three waves of friluftsliv thought (see Figure 1, below). In our view, the first was from the 1850s to 1960s, where the term “friluftsliv” was being used explicitly, but only by a few educated souls. What we might recognize as friluftsliv activities, such as foraging for food in the forest, or hiking and skiing on trails, were increasingly widespread. For some people, at least, using the forest or sea as a workplace or for gathering food for the family was considered friluftsliv.

A second wave came in the late 1960s, which was based on a logic of deep ecology and environmental sustainability, and which attempted to put clear boundaries around the attitudes and activities that characterized “proper” friluftsliv. The last 25 years or so could be considered a third wave of friluftsliv, where these boundaries have loosened and become more ambiguous, in order to reflect and include a society that is at once becoming very multicultural and imbued with mobile phones, social media, and advanced clothing and equipment.

While friluftsliv has always featured a certain amount of subjectivity [see (5)], it seems we have moved towards a kind of friluftsliv that is more subjective, personal, and fluid. In this view, there is not one idealized form of friluftsliv and not one “right” way to practice it. For some, friluftsliv is going for a 50 km ski through the forest, using all of the latest clothing and equipment; for others, it may be a family barbecue around a firepit by a lake that is a hundred metres from a parking lot. At its most fundamental basis, friluftsliv thus becomes “anything an individual wants it to be”, as long as it is time that people choose to spend outdoors and which brings them feelings of well-being. This openness resonates with the Norwegian government's definition that was presented early on in this paper (6). Indeed, as Horgen (50) notes, “Norwegian friluftsliv is continually evolving, interplaying with the surrounding society, and is far from the static, unchanging construct some might envision” (p. 38).

This paper offers four principal contributions to the literature. First, the English language friluftsliv literature (largely authored by Scandinavians and North Americans) has depicted a certain romantic and idealized conception of friluftsliv. Second, three historical waves of friluftsliv are presented, which illustrate the changing nature of meanings associated with it over time. Third, the paper builds on the growing acceptance that friluftsliv can be difficult to define and increasingly means many things to many people. Fourth, it is highlighted how friluftsliv—just as outdoor education, leisure and tourism in other parts of the world—needs to address challenges relating to inequalities, over-consumption, and increased carbon emissions. Contemporary *friluftsliv* practices arguably have much in common with nature-based leisure in many other countries, as they encompass an increasingly wide range of activities through which a diverse population connects with nature in personally meaningful ways.

## Data Availability

The original contributions presented in the study are included in the article/Supplementary Material, further inquiries can be directed to the corresponding author.
